# Activation of HIV-1 with Nanoparticle-Packaged Small-Molecule Protein Phosphatase-1-Targeting Compound

**DOI:** 10.3797/scipharm.1502-01

**Published:** 2015-06-22

**Authors:** Kahli A. Smith, Xionghao Lin, Oleg Bolshakov, James Griffin, Xiaomei Niu, Dmytro Kovalskyy, Andrey Ivanov, Marina Jerebtsova, Robert E. Taylor, Emmanuel Akala, Sergei Nekhai

**Affiliations:** 1 Center for Sickle Cell Disease, Howard University, Washington DC 20059, USA; 2 Department of Pharmacology, Howard University, Washington DC 20059, USA; 3 Department of Pharmaceutical Sciences, Howard University, Washington DC 20059, USA; 4 College of Engineering, Howard University, Washington DC 20059, USA; 5 Department of Biochemistry, School of Medicine, University of Texas Health Science Center at San Antonio, 7703 Floyd Curl Dr., San Antonio TX 78229, USA; 6 Enamine LLC, Princeton Corporate Plaza, 7 Deer Park Drive, Ste. M-3 Monmouth Jct., NJ 08852, USA; 7 Department of Microbiology, College of Medicine, Howard University, Washington DC 20059, USA; 8 Department of Medicine, Howard University, Washington DC 20059, USA

**Keywords:** HIV-1, Transcription, Latency, Nanoparticles, Small molecules, PP1, SMAPP1

## Abstract

Complete eradication of HIV-1 infection is impeded by the existence of latent HIV-1 reservoirs in which the integrated HIV-1 provirus is transcriptionally inactive. Activation of HIV-1 transcription requires the viral Tat protein and host cell factors, including protein phosphatase-1 (PP1). We previously developed a library of small compounds that targeted PP1 and identified a compound, SMAPP1, which induced HIV-1 transcription. However, this compound has a limited bioavailability *in vivo* and may not be able to reach HIV-1-infected cells and induce HIV-1 transcription in patients. We packaged SMAPP1 in polymeric polyethylene glycol polymethyl methacrylate nanoparticles and analyzed its release and the effect on HIV-1 transcription in a cell culture. SMAPP1 was efficiently packaged in the nanoparticles and released during a 120-hr period. Treatment of the HIV-1-infected cells with the SMAPP1-loaded nanoparticles induced HIV-1 transcription. Thus, nanoparticles loaded with HIV-1-targeting compounds might be useful for future anti-HIV-1 therapeutics.

## Introduction

Anti-Retroviral Therapy (ART) has been very effective in the inhibition of HIV-1 replication, but it cannot eradicate the virus completely due to the existence of latently infected cells, which are not accessible or susceptible to the existing antiretroviral drugs [1]. The integrated HIV-1 provirus is not affected by the existing anti-HIV-1 drugs unless its transcription is activated [2]. Recently, pharmaceutical approaches have focused on the development of activators of latent HIV-1. HIV-1 transcription is induced by the Tat protein that recruits CDK9/cyclin T1 to TAR RNA (reviewed in [3]). CDK9/cyclin T1 activity is regulated by the association/dissociation with the 7SK small nuclear RNA ribonuclear protein (7SK snRNP) complex which involves reversible CDK9 phosphorylation and dephosphorylation [3]. Qiang Zhou’s lab showed that CDK9 Thr 186 is dephosphorylated by protein phosphatase-1 (PP1) which helps to dissociate CDK9/cyclin T1 from 7SK RNP [4]. We also showed that PP1 sequestration through stable expression of the central domain of the nuclear inhibitor of PP1 (cdNIPP1) led to the accumulation of CDK9 in 7SK snRNP and inhibition of HIV-1 [5]. We also showed that PP1 dephosphorylates the CDK9 Ser 175 residue [6]. In a recent study, Jonathan Karn and colleagues showed that the CDK9 Ser 175 residue is phosphorylated in activated T cells and that this phosphorylation promotes the interaction of CDK9/cyclin T1 with Tat and activation of HIV-1 transcription [7]. Jonathan Karn and colleagues also showed that the CDK9 Ser175A mutant efficiently reactivated the latent HIV-1 provirus which compares to WT CDK9 or CDK9 S175D because of the inability of the CDK9 S175A mutant to bind to BRD4 [7]. Thus, PP1 can contribute to HIV-1 transcription activation by dissociating CDK9/cyclin T1 from 7SK snRNP and preventing CDK9 scavenging by BRD4. This can be followed up by CDK9 Ser175 phosphorylation which will facilitate CDK9/cyclin T1 binding to HIV-1 Tat and prevent reentry of CDK/cyclin T1 to 7SK snRNP [7]. PP1 is a dimer of one of the three catalytic subunits and one of the multiple regulatory subunits. The catalytic subunit of PP1 contains an “RVxF”-accommodating cavity where it binds the “RVxF” sequence containing regulatory subunits including Tat’s QVCF sequence [8]. HIV-1 Tat translocates PP1 to the nucleus and disruption of this binding and nuclear translocation prevents Tat-mediated induction of HIV-1 transcription [8]. We recently developed a library of small molecules that targeted the non-catalytic RVxF-binding site of PP1 [9, 10]. From this library, we identified several compounds that induced HIV-1 transcription [11]. The best compound, the small-molecule activator of PP1 (SMAPP1) induced PP1 *in vitro* and activated HIV-1 transcription in acutely and chronically infected T cells [11]. Here we analyzed the stability of SMAPP1 in water solution and plasma and demonstrated that the compound is quickly degraded in plasma. Therefore, the low stability of SMAPP1 may limit bioavailability *in vivo* and will require frequent administration in patients which may result in low patient compliance and treatment cessation. In this study, we have developed an innovative polyethylene glycol polymethyl methacrylate (PEG PMMA) nanoparticle SMAPP1 delivery system and characterized nanoparticles. We demonstrated that SMAPP1-containing nanoparticles activated HIV-1 in infected cultured cells. Our results demonstrate that nanoparticle-packaged small molecules can activate HIV-1 transcription and provide proof-of-principle experiments showing how novel delivery systems may enhance future HIV therapy.

## Materials and Methods

### Materials

Methacryol chloride, methyl methacrylate, hydroxylamine hydrochloride, pyridine, diethyl ether, benzoyl peroxide (BP), *N*-phenyldiethanolamine (NPDEA), and acetonitrile were purchased from Sigma–Aldrich. Ethyl alcohol and hydrochloric acid were obtained from Fisher Scientific. Poly(ethylene glycol)*n* monomethyl ether monomethacrylate (PEG-MA, *n* = 1000) was purchased from Polysciences Inc. Small Molecular Activator of PP1 (SMAPP1) was synthesized by ChemoBioCenter (Kiev, Ukraine).

### Nanoparticle Preparation

**Fig. 1:**
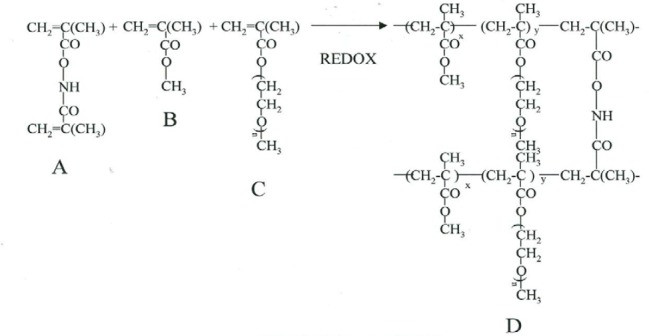
Synthesis of stealth hydrolyzable crosslinked PEG-PMMA nanoparticles (A = N,O-dimethacryloyl hydroxylamine (XL); B = methyl methacrylate (MMA); C = poly(ethylene glycol)n monomethyl ether monomethacrylate (PEG-MA; n=1000); D = crosslinked polymer)

The dispersion polymerization technique was used for the fabrication of the nanoparticles. Free radical generation was by benzoyl peroxide (BP) and N-phenyldiethanolamine (NPDEA) (used as an initiator system). The synthetic scheme for production of the hydrolysable crosslinked PEG PMMA nanoparticles is shown on Fig. 1. The synthesis of the polymeric nanoparticles proceeded by the free radical dispersion polymerization of a hydrophilic macromonomer (poly(ethylene glycol) monomethyl ether monomethacrylate (PEG-MA)) and a hydrophobic comonomer (methyl methacrylate (MMA)) in a binary solvent system (ethanol:water) crosslinked by N,O-dimethylacryloyl hydroxylamine. The mechanism for the formation of the polymeric nanoparticles synthesized by dispersion polymerization has been reported previously in publications [12–15].

Briefly, a binary solvent system ethanol:water (10 mL), MMA (4.0 mmol), PEG-MA (0.1 mmol), XL (0.162 mmol), and co-initiators: BP and NPDEA (1:1) (1% of the polymerizable components) were mixed in a round-bottomed flask with constant stirring. The drug-loading was carried out by adding a fixed amount of the compound (10 mg of SMAPP1). The polymerization was performed overnight with continuous flow of nitrogen gas. After the polymerization, the resulting product was centrifuged at 10,000 RPM for 25 minutes to collect the solid particles. The pellet was dispersed in 2 mL of distilled water, freeze–dried, and stored in vials at 4°C. The average weight of the nanoparticles (the amount of nanoparticles recovered after freeze-drying) was 15.8 mg.

### Synthesis and Characterization of Hydrolyzable Crosslinker (N,O-dimethylacryloyl hydroxylamine)

The crosslinker (XL), *N*,*O*-dimethacryloyl hydroxylamine, was synthesized as described previously [12, 13, 16], and was characterized by ^1^H-NMR analysis and melting point determination. The data obtained {m.p.: 55–56°C; ^1^H-NMR (400 MHz, CDCl_3_): (C_8_H_11_NO_3_) δ = 2.04 (s, 3H), 2.08 (s, 3H) 5.54, 5,8 (s, 2H), 5.9, 6.38 (s, 2H), 9.25 (s, 1H)} were in agreement with previously obtained data.

### High-Performance Liquid Chromatography

High-performance liquid chromatography (HPLC) was used for the *in vitro* drug release study. The Hewlett-Packard (HP) liquid chromatography system Series 1100 equipped with a C18 reversed-phase Zorbax (300SB) HPLC column (5 μm; 4.6 x 250 mm) and an HP photodiode array (DAD) detector was used. The peak of UV absorbance for SMAPP1 was determined by UV/Vis spectrophotometry and found to be at 245 nm (data not shown). Thus, the wavelength 245 nm was used to detect and monitor the release of SMAPP1 from nanoparticles. The elution gradient started with 50% acetonitrile: 50% H_2_O and it was changed linearly to 100% acetonitrile for 8 min. The flow rate was 1 mL/min.

### Scanning Electron Microscopy (SEM)

Different dilutions of the nanoparticle suspension in distilled water were placed on carbon tape affixed to an aluminum stub (SPI Supplies, Inc.) and dried in a vacuum oven for 24 hours. The samples were gold-coated with a Hummer Sputtering machine for 2 minutes under an argon atmosphere. The samples were then observed using a JEOL JSM 7600F scanning electron microscope. A 5kV accelerating voltage and secondary electron mode was used with a working distance of 12 mm for the morphological characterization. The images were taken at different magnifications ranging from 10000 to 200000x.

### SMAPP1 Stability Assay

SMAPP1 dissolved in DMSO (10 mM) was mixed with mouse serum to the final concentration of 100 hM and incubated at 37°C. Aliquots were collected at different time points up to 24 hrs. In addition, CEM T cells were seeded in 24-well plates (100,000 cells/well) and treated with SMAPP1 (10 µM) for 12 hrs. Cell culture media was collected at different time points over 48 hrs and the total protein was precipitated by the addition of four volumes of cold acetone and centrifugation at 10,000xg for 15 min. The supernatant was collected and evaporated to dryness using a SpeedVac concentrator. The dry pellet was reconstituted in acetonitrile, and 10 el aliquots were injected into the nanocolumn for LC-MS analysis. The LC-MS system consisted of a Shimadzu nano LC coupled with an LTQ Orbitrap XL tandem mass spectrometer (Thermo Fischer Scientific, GA, USA). The mobile phase consisted of a 0.1% formic acid aqueous solution (A) and a 0.1% formic acid acetonitrile solution (B). The gradient elution program was as follows: 0–6.02 min, 1% B; 6.02–6.11 min, 1–2% B; 6.11–20 min, 2−80% B; 20–25 min, 80% B; 25–30 min, 80–85% B; 30–31 min, 80–2% B; 31–40 min, 2% B (v/v). The flow rate was set at 600 nl/min. The compounds were ionized by electrospray ionization and detected by Orbitrap at 30000 mass resolution (full scan, *m/z* 150−2000). The spray voltage, capillary temperature, and capillary voltage were set to 2.0 kV, 200°C, and 39.5 V, respectively.

### Cells

CCRP-CEM (CEM, CEM T) cells were purchased from ATCC (Manassas, VA) and maintained in RPMI media supplemented with 10% fetal bovine serum (FBS) and streptomycin-penicillin (all from Invitrogen) at 37°C and 5% CO_2_.

### VSVG-HIV-Luc Infection

VSVG HIV-*Luc*-pseudotyped HIV-1 virus was prepared as described previously [17]. CEM T cells were infected with the VSVG HIV-*Luc* virus and incubated overnight. The infected cells were then treated with either PP1-targeting compounds or nanoparticles and incubated overnight. Both uninfected cells and cells infected with the pseudotyped virus were used as negative and positive controls, respectively. The luciferase lysis buffer (SteadyLite, Perkin Elmer) was added to the cells and luminescence was measured using the Luminoskan (Perkin-Elmer).

### Cellular Toxicity

Cellular toxicity was measured using the Calcein-AM assay (R&D Systems). CEM T cells were treated in a white 96-well plate with empty nanoparticles and nanoparticles packaged with SMAPP1 overnight. DMSO was used as a control. Cells were treated with Calcein-AM and fluorescence was measured according to the manufacturer’s instructions.

## Results

### SMAPP1 Activates HIV-1 Transcription

**Fig. 2:**
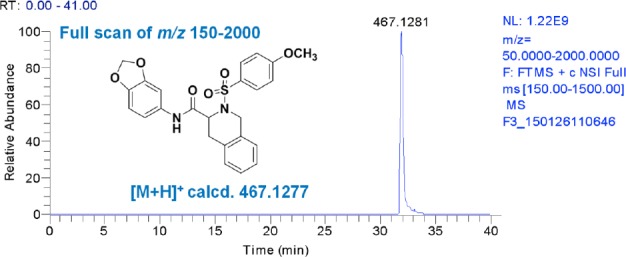
Mass spectrometry analysis of SMAPP1

We developed and characterized a small-molecule, 1E7-03 with an acridine scaffold, that targeted a non-catalytic site of PP1 and inhibited HIV-1 transcription and replication [9]. We then substituted the acridine scaffold with an isoquinoline scaffold and produced a novel library containing 38 new compounds. Screening of this library using single-round HIV-1 replication in CEM T cells infected with the VSVG-pseudotyped HIV-1 virus discovered an effective activator of HIV-1 transcription, named the small-molecule activator of PP1 (SMAPP1) (Fig. 2) [11, 18].

**Fig. 3:**
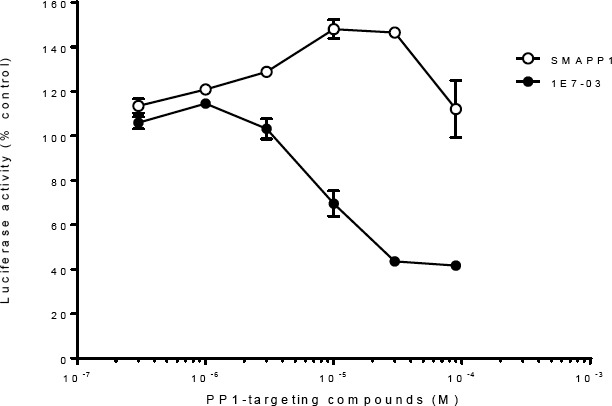
HIV-1 transcription activation by SMAPP1

In contrast to 1E7-03 that inhibited HIV-1 transcription [9], SMAPP1 increased transcription by about 50% in single-round HIV-1 infection at the concentration 10–100 µM (Fig. 3).

**Fig. 4:**
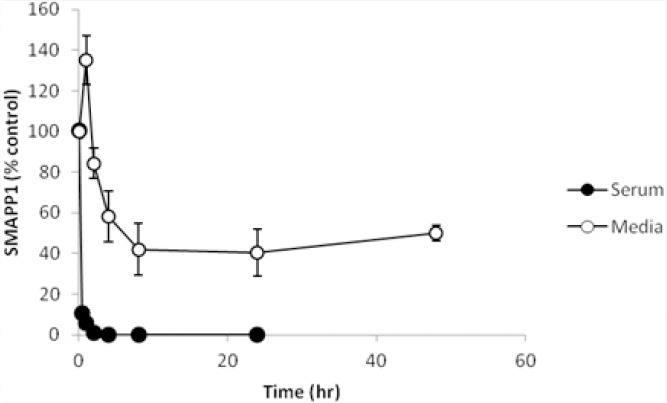
SMAPP1 stability in RPMI media and serum

SMAPP1 was relatively stable in the cell culture media with 50% degradation over 5 hrs, but rapidly degraded in serum (Fig. 4).

### Fabrication of SMAPP1 Containing PEG-PMMA Nanoparticles

**Fig. 5:**
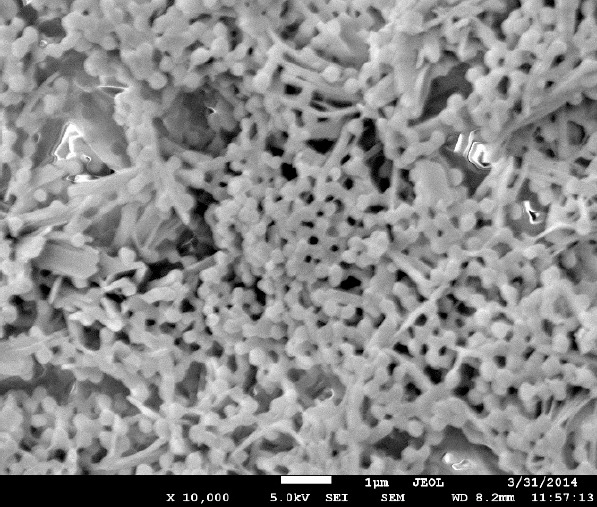
Scanning electron micrograph (SEM) of SMAPP1-loaded PEG-PMMA nanoparticles. Original magnification 10,000 x

To increase the stability and bioavailability of SMAPP1, we packaged it into nanoparticles. The dispersion polymerization technique was used for the fabrication of the nanoparticles. The mechanism for the formation of the polymeric nanoparticles synthesized by dispersion polymerization was reported previously [12, 13] and described in detail in “Materials and Methods.” Formation of the nanoparticles was confirmed by scanning electron microscopy (SEM) analysis (Fig. 5). The image shows well-formed, monodispersed nanoparticles with a narrow particle size distribution (not shown) which appear as aggregates of spheres joined together by the surface of polyethyleneglycol.

### Loading Capacity of Nanoparticles

**Fig. 6:**
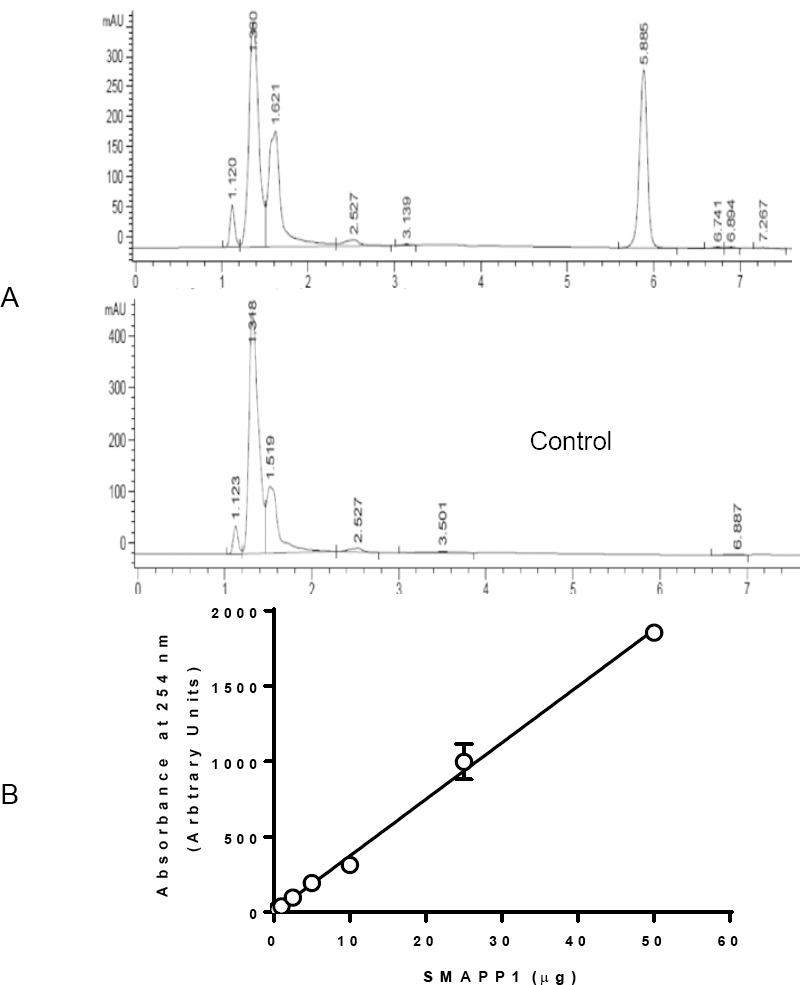
A: HPLC profile of SMAPP1 in RPMI media and media control; B: calibration curve for SMAPP1, N=3.


A fixed amount of SMAPP1-loaded nanoparticles were incubated in the RPMI cell culture media buffer (pH 7.4) for 30 days till all of the SMAPP1 was released. The amount of SMAPP1 in the buffer was determined by HPLC (Fig. 6A) as described in “Materials and Methods” using a calibration curve (Fig. 6B). The encapsulation property of the nanoparticles can be expressed as loading capacity (amount of the drug encapsulated expressed as a percentage of the nanoparticle weight) or entrapment efficiency, which is the percentage of the initial drug in the encapsulation solution that is entrapped [19] While drug-loading can be expressed as loading capacity or encapsulation efficiency for nanoparticles fabricated from prepolymers, loading capacity is appropriate for the nanoparticle network made by in situ polymerization [20]. The loading capacity of the nanoparticles was calculated by the following equation:

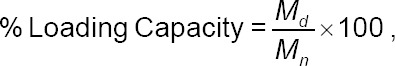


where *M_d_* is the weight of SMAPP1 in a known weight of the nanoparticle network (*M_n_*). The percent loading capacity of nanoparticles containing SMAPP1 was 0.8%.

### In Vitro Drug Release

**Fig. 7:**
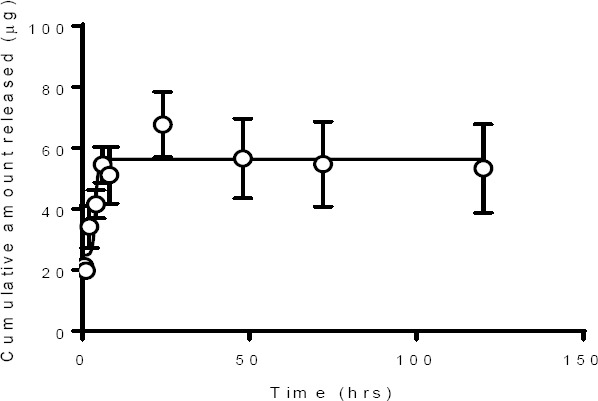
SMAPP1 release from PEG-PMMA nanoparticles

To determine the stability of SMAPP1 in nanoparticles and the kinetics of SMAPP1 release, the drug release studies were performed by the dialysis method. SMAPP1-loaded nanoparticles (10 mg) were placed in a dialysis bag with a 12,000–14,000 Da cutoff (Spectra/Por^®^CE) containing 2 ml of PBS buffer (pH 7.4) and placed in the tube which contained 20 ml of buffer. The tube was clamped onto a Labquake Tube Shaker (Fisher Scientific) capable of 360 degrees rotation and incubated at 37°C with rotation. Aliquots of 2 ml were taken at different time points (from 0.5 to 144 hours), and the volume was replaced with 2 ml of fresh buffer. The solution was filtered through a 0.2 mm filter, analyzed by HPLC, and the amount of released SMAPP1 was calculated using a standard curve (Fig. 6B). SMAPP1 was released from PEG-PMMA nanoparticles during the first 24 hrs and the release continued for up to 120 hrs (Fig. 7).

### SMAPP1-Loaded Nanoparticles Activate HIV-1 Transcription

**Fig. 8:**
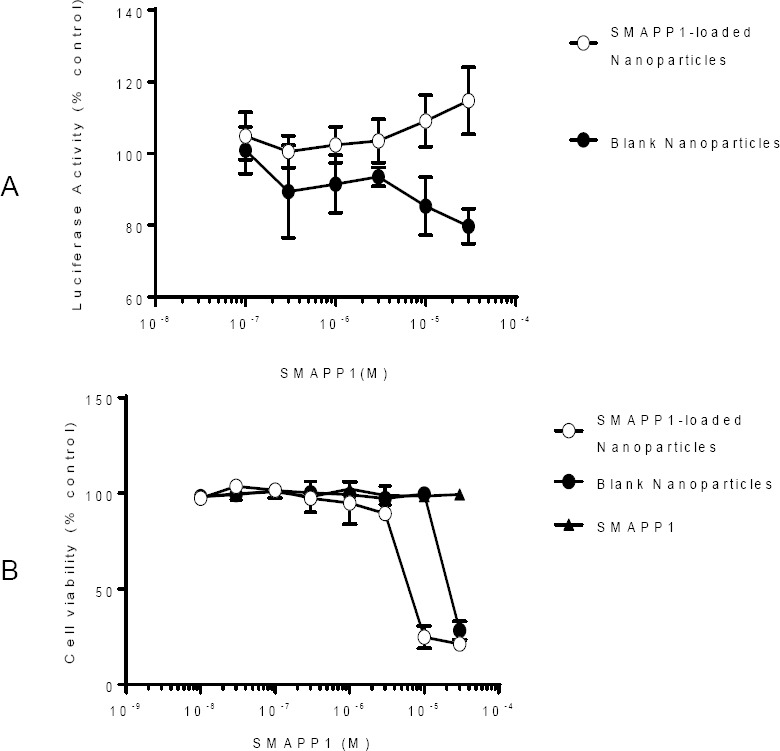
A: Effect of SMAPP1-loaded PEG-PMMA nanoparticles on HIV-1 transcription; B: cell toxicity.

We next analyzed the effect of SMAPP1-loaded nanoparticles on a single round of HIV-1 replication in CEM T cells infected with VSVG-pseudotyped HIV-1 virus expressing luciferase (HIV-1 *Luc*). Overnight treatment of the infected CEM T cells with SMAPP1-loaded nanoparticles at 1–10 µM concentration induced HIV-1 transcription by about 50% (Fig. 8A). While SMAPP1 showed no significant effect on cell viability, SMAPP1-loaded nanoparticles reduced cell viability by 30–50% at 10 µM concentration and significantly reduced cell viability at higher concentrations (Fig. 8B). Taken together, these results indicate that SMAPP1-loaded PEG-PMMA nanoparticles can induce HIV-1 gene expression in infected T cells.

## Discussion

Elimination of latent HIV-1 reservoirs is extremely challenging because these reservoirs are established early in HIV-1 infection, they are stable for a long time, and they can be replenished during episodes of viremia. Latently infected cells do not express HIV-1 proteins or express only minimal levels of viral proteins and thus cannot be easily recognized and eliminated by the immune system or targeted by antiretroviral drugs. Several pathways and transcription complexes regulate the activity of the HIV-1 promoter and might serve as molecular targets for compounds to activate latent HIV-1 [21]. Activation of CDK9, which can stimulate viral transcription and enhance efficiency of transcriptional elongation, represents a possible route for the activation of the latent virus. Recently, bromodomain (BET) family inhibitors (JQ1 and prostratin) were reported to reverse HIV latency in latently HIV-1-infected cell lines and in several primary cell models of HIV latency [22–24]. Two BET family members (BRD4 and BRD2) have been shown to bind CDK9 complexes and serve as cofactors for the Tat protein [7, 24, 25]. We recently found that the small PP1 binding molecule (SMAPP1) activates latent HIV-1 (Tyagi and Nekhai, manuscript under revision). The low solubility of SMAPP1 in water solutions and low stability in serum represent major obstacles for its potential use as therapeutics.

Recently drug-loading nanoparticle delivery systems have been explored in HIV antiretroviral research. They offer several advantages like the enhancement of bioavailability, water solubility, stability, and targeting ability. Liposomes, nanoparticles, niosomes, polymeric micelles, and dendrimers have been used for packaging HIV-1 inhibitors [26]. However, the study of nanoparticles for the activation of HIV latency is very limited. Development of lipid nanoparticles loaded with the protein kinase C activator bryostatin-2 that stimulated the latent virus in human T cell lines *in vitro* and in a humanized mouse model *ex vivo* has been recently demonstrated [27]. Lipid nanoparticles have also been loaded with both bryostatin-2 and the protease inhibitor nelfinavir, producing particles capable of both activating the latent virus and inhibiting viral spread. These data demonstrate the ability of nanotechnological approaches to provide improved methods for activating latent HIV infections.

Polymer conjugation is a widely used technique for the improvement of drug therapeutic properties. Polymer-conjugated drugs exhibit prolonged half-life, higher stability, and water solubility [28]. Poly(ethylene glycol) (PEG) has been approved by the FDA for several medical applications because of its biocompatibility and low toxicity. Here we described the SMAPP1-loaded PEG-PMMA nanoparticle preparation to increase its bioavailability. The loading capacities of the SMAPP1-loaded nanoparticles were low (0.8%), but can be improved.

The release of SMAPP1 from nanoparticles is slow and extends for a long period of time (more than 24 h) which is likely due to the hydrolysis of the crosslinked core and release of the drug encapsulated within the matrix of the nanoparticles. The hydrolyzable crosslinker in the nanoparticle matrix is susceptible to hydrolysis at pH 7.4 [29, 30].

Both loaded and unloaded nanoparticles demonstrated cell toxicity at a concentration higher than 10 µM. SMAPP1-loaded nanoparticles demonstrated a higher toxicity than unloaded nanoparticles despite the absence of toxicity of SMAPP1. One of the possible explanations is that at high concentrations (>10 µM), SMAPP1 was not soluble in the cell media; while nanoparticles increased its *in vitro* bioavailability via internalization by endocytosis and consequently, made it more toxic for cells.

## Conclusion

SMAPP1-loaded PEG-PMMA nanoparticles were successfully formulated by the dispersion polymerization technique. SMAPP1 demonstrated sustained release for more than 24 hrs which may help to reduce multiple daily doses to once per day. SMAPP1-loaded nanoparticles activated HIV-1 transcription in cell cultures.
